# Convention of Medical Delegates

**Published:** 1851-10

**Authors:** 


### CONVENTION OF MEDICAL DELEGATES.

We are pleased to learn that the indications of a large attendance of delegates to the proposed convention for constituting a State Medical Society, in Kentucky, are in the highest degree propitious to the success of the enterprize. It is singular that a State which has made so many contributions to the progress of medical science, as Kentucky has, which, for a long period of time, had the principal medical school of the west and south in it, should have been so long without a State Medical Society. But the profession is awaking to the importance of having such a society, and we earnestly hope that Kentucky will soon possess one, fixed upon a proper basis for usefulness, devoted to no clique or party, but to the advancement and elevation of the whole profession, and each worthy member of it. It is within the capacity of the physicians of Kentucky, to found a medical society that may vie with any in the Union in all the elements of usefulness and permanent prosperity, and sincerely do we trust that such will be the result of the labors of the convention.

